# Clinical presentation, risk factors, and comorbidities of cutis marmorata telangiectatica congenita: a systematic review

**DOI:** 10.1038/s41390-025-04588-8

**Published:** 2025-12-09

**Authors:** Isabella Kamholtz, Simonetta I. Gaumond, Lawrence A. Schachner, Joaquin J. Jimenez

**Affiliations:** 1 Dr. Phillip Frost Department of Dermatology and Cutaneous Surgery, University of Miami Miller School of Medicine, Miami, FL USA; 2 Department of Biochemistry and Molecular Biology, University of Miami Miller School of Medicine, Miami, FL USA

## Abstract

**Background:**

Cutis marmorata telangiectatica congenita (CMTC) is a rare vascular disorder characterized by reticulated erythematous or violaceous patches, often accompanied by complications such as ulceration, atrophy, and limb asymmetry. Despite its rarity, CMTC poses diagnostic and prognostic challenges, necessitating a clearer understanding of its clinical course and associated comorbidities.

**Methods:**

A systematic literature search was conducted using PubMed to identify case reports and case series published in the last five years. Inclusion criteria encompassed studies reporting clinical findings, associated comorbidities and long-term outcomes of CMTC. Data extraction focused on lesion characteristics, anatomical distribution, associated complications, and comorbidities.

**Results:**

Nineteen articles were identified, encompassing 22 cases ranging from neonates to adolescents. Most lesions were localized, primarily affecting the lower extremities (55%), while 41% exhibited generalized involvement. Lesions remained stable or improved over time. Complications included atrophy (41%), limb asymmetry (32%), and ulceration (18%). Associated comorbidities were reported in 50% of cases, with ophthalmologic anomalies, such as retinal nonperfusion and neovascularization, being the most common.

**Conclusion:**

The limited availability of clinical trials and longitudinal follow-up data highlights significant gaps in understanding CMTC’s long-term progression and outcomes. Further robust longitudinal studies are necessary to enhance clinical management and prognostic guidance for affected patients.

**Impact statement:**

- Cutis marmorata telangiectatica congenita (CMTC) is a rare congenital vascular disorder with variable presentation and frequent comorbidities.
- This systematic review synthesizes recent pediatric cases, highlighting key features such as limb asymmetry, atrophy, and ulceration, and identifying ophthalmologic involvement as the most common complication.
- By underscoring the high rate of comorbidities and the psychosocial impact of visible lesions, our study reinforces the need for multidisciplinary care.
- It also identifies gaps in long-term outcome data and standardized management, providing a foundation for future prospective research to improve clinical recognition, prognostic accuracy, and patient care.

**Highlights:**

- This systematic review synthesizes recent case reports and series of CMTC, defining its variable clinical features, risk factors, and comorbidities, emphasizing the need for multidisciplinary care.
- It provides the most up-to-date summary of CMTC within the past five years, identifying ophthalmologic anomalies as the most frequent comorbidity and underrecognized features such as limb asymmetry and lesion stability.
- This systematic review exposes critical gaps in long-term follow-up and standardized management, emphasizing the need for prospective studies.
- Findings support routine ophthalmologic screening and individualized care in pediatric patients, even in the absence of systemic symptoms.

## Introduction

Cutis marmorata telangiectatica congenita (CMTC) is a rate congenital vascular malformation first described by Dr. Cato van Lohuizen in 1922.^1^ It presents as persistent reticulated erythematous or violaceous patches at birth, which may fade within the first two years of life.^2,3^ Unlike physiological cutis marmorata, CMTC lesions do not resolve with warming and may be associated with atrophy, ulceration, and limb asymmetry, potentially affecting function and quality of life.^2,3^

**Fig. 1:**
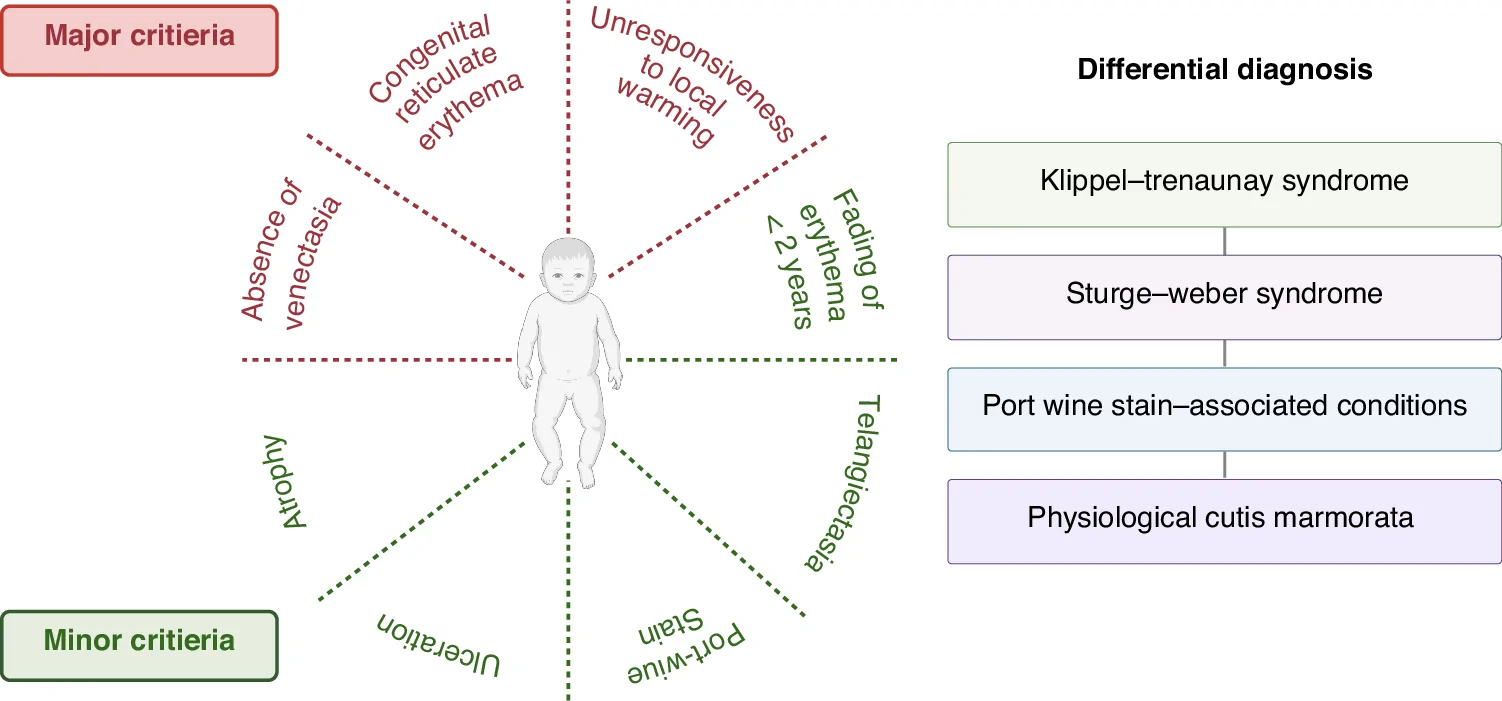
Diagnostic Criteria and Differential Diagnosis.

Diagnosing CMTC can be challenging due to this overlap with other vascular anomalies, such as Klippel-Trenaunay syndrome, Sturge-Weber syndrome, port wine stain-associated conditions, and physiological cutis marmorata.^4–6^ Additionally, the term *Macrocephaly-CMTC Syndrome* is now considered a misnomer, as the vascular findings in these patients often do not represent true CMTC but rather features of capillary malformations, such as port wine stains or persistent cutis marmorata.^7^ In light of these distinctions, experts have proposed renaming the condition *Macrocephaly-Capillary Malformations* to more accurately reflect the underlying vascular anomalies. To improve diagnostic accuracy, *Kienast & Hoeger*^3^ proposed clinical criteria requiring three major features (congenital reticulate erythema, absence of venectasia, and unresponsiveness to warming) and at least two minor criteria (e.g., telangiectasia atrophy, or port-wine stains) (Fig. 1). Increased clinical awareness is essential to differentiate CMTC from mimickers and prevent delays in diagnosis.

Despite its distinct clinical presentation. CMTC remains understudies, with most literature limited to case reports and small series. Long-term outcomes, associated comorbidities, and optimal management strategies are poorly defined. This review aims to summarize current knowledge on CMTC, including clinical presentation, risk factors, prognosis, and comorbidities, to enhance recognition and improve patient care.

## Methods

A systematic literature search was conducted using PubMed to identify case reports and case series on CMTC published in the past five years. The search included the terms: *CMTC*, *Van Lohuizen syndrome*, *nevus vascularis reticularis*, *congenital generalized phlebectasia*, *congenital phlebectasia*, *livedo telangiectatica*, and *congenital livedo reticularis*. Articles were included if they were published in English, focused on CMTC, and provided relevant clinical details.

**Fig. 2:**
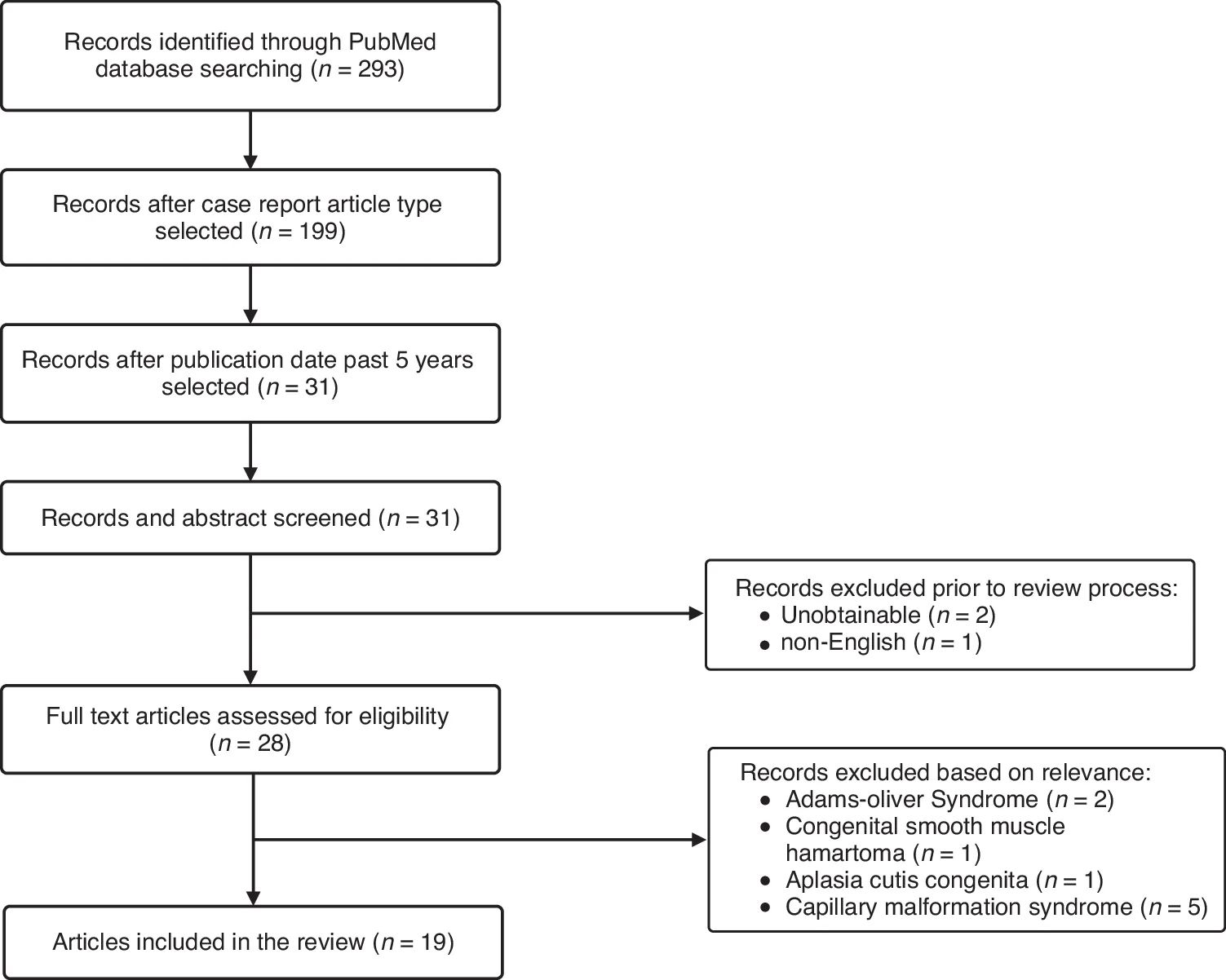
Literature Search Flowchart.

The search yielded a total of 31 case studies, of which 19 met inclusion criteria after screening titles, abstracts, and full texts. Articles were excluded if they lacked accessible full-text manuscripts (*n* = 2), were outside the scope of the review (*n* = 9), or were non-English publications (*n* = 1) (Fig. 2).

Extracted data included patient demographics, clinical presentation, lesion distribution patterns, ulceration or atrophy, limb asymmetry, comorbidities (categorized as ophthalmologic, cutaneous, or systemic), and treatment response. Environmental and physiological factors influencing lesion characteristics were recorded. Findings were synthesized descriptively, with key features summarized in tables for clinical reference. Risk of bias analysis was completed using the Joanna Briggs Institute (JBI) critical appraisal checklists for case reports and case series.^8,9^ A generalized analysis is presented in Table 1, with supplemental information provided in Supplementary tables S1 and S2.

**Table 1:** Quality rating scheme for studies using the JBI checklist.

Study type	Study	JBI rating	Quality rating	Reference
Case series	Leung et al., 2020	5/10	Moderate	^11^
	Moreno-Alfonso et al., 2024	7/10	High	^12^
	Shah et al., 2024	6/10	Moderate	^20^
Case reports	Portal Buenaga et al., 2024	3/8	Low	^10^
	Kyriakou et al., 2020	5/8	High	^13^
	Manna, 2024	5/8	High	^14^
	Yang et al., 2023	5/8	High	^15^
	Cardis et al., 2020	4/8	Moderate	^16^
	MacGibeny et al., 2020	5/8	High	^17^
	Ghotbabadi et al., 2024	5/8	High	^18^
	Kim and Kim, 2022	5/8	High	^19^
	Hokazono et al., 2024	5/8	High	^21^
	Rosignoli et al., 2022	4/8	Moderate	^22^
	Khambati et al., 2021	4/8	Moderate	^23^
	Tracey et al., 2020	4/8	Moderate	^24^
	Friedman et al., 2023	5/8	High	^25^
	Belgemen-Ozer, 2024	5/8	High	^26^
	Haidari et al., 2020	5/8	High	^27^
	Sassalos et al., 2021	5/8	High	^28^

## Results

Twenty-two cases were reviewed, which included patients ranging from neonates (0–28 days) to adolescents (age 16). Neonates comprised eight cases,^10–17^ followed by six cases of infants (1 month–1 year),^18–23^ and four cases of toddlers (1–5 years).^12,20,24,25^ Other cases included a ten-year-old,^26^ a 15-year-old,^27^ and a 16-year-old.^28^ The majority were female, with male patients accounting for 45%. Two patients reported a family history of vascular anomalies.^14,27^

### Clinical presentation

The clinical presentation of CMTC in this cohort was variable, with differences observed in distribution pattern, ulceration, atrophy, and limb asymmetry.

#### Distribution

Seventeen cases involved the lower extremities, and seven involved the upper extremities. Nine patients (41%) exhibited a generalized distribution of CMTC lesions,^10,13,15,18–21,26,28^ with one of these cases involving mucous membranes^15^ (Table 2). In contrast, 12 patients (55%) had localized lesions. Nine had lesions confined to the lower extremity.^11,12,14,16,17,23,24^ Two patients had lesions localized to the back, specifically with one having CMTC on the lower back,^27^ and the other on the upper back.^22^ One patient had lesions present only on the neck,^20^ and distribution was not discussed in another.^25^

**Table 2:** Summary of results.

**Clinical features**	**Feature**	**Description**	**Prevalence in cases reviewed**
	Distribution pattern	Localized vs. generalized lesions	Localized: 55% Generalized: 41%
	Ulceration	Presence of ulceration within lesions	18%
	Atrophy	Thinning of skin in affected areas	41%
	Limb asymmetry	Length or circumference discrepancies	32%
	Environmental responses	Changes with cold, exercise, or fever	50%
**Risk factors and prognosis**	Temperature	Cold exposure exacerbates lesions	9%
	Physiological changes	Exacerbation with crying, fever, or exercise	14%
	Resolution over time	Spontaneous fading within 2 years	14%
	Intervention outcomes	Improvement with pulsed dye laser therapy	5%
**Comorbidities**	Ophthalmologic	Neovascularization, retinal nonperfusion	27%
	Cutaneous	Hemangiomas, mastocytoma	9%
	Systemic	Liver function abnormalities, Rubinstein-Taybi Syndrome, localized edema	14%

#### Ulceration and atrophy

Four patients (18%) had an ulceration within CMTC lesions, with some patients having one ulcer^13,27^ and others having two^24^ or three^17^ (Table 2). Nine patients (41%) had atrophy, of which eight had CMTC involvement on a lower extremity.^11,12,16,17,21,24^ One patient had atrophy in the temporal region above the coronal suture.^13^ Three patients (14%) had both atrophy and ulceration.^13,17,24^

#### Limb asymmetry

Seven patients (32%) had some degree of limb asymmetry, either hypertrophy or hypotrophy, associated with CMTC (Table 2).

One patient had a lower leg length discrepancy, with the affected leg measuring 1.5 cm less than the contralateral leg.^14^ Four patients had a circumference discrepancy, with three reporting the CMTC-affected limb as smaller.^10,16,24^ This included a thigh circumference difference of 1.5 cm in one patient.^24^ One patient had a circumference discrepancy due to hypertrophy, where the CMTC-affected arm and leg were larger by 1 cm and 1.5 cm, respectively.^26^

Two patients had both length and circumference discrepancies, with the CMTC-affected limb being shorter and having smaller circumferences.^11^ In the first case, the CMTC-affected limb was 2.5 cm shorter and had circumference differences of 3.3 cm for the midthigh and 2.6 cm for the midcalf. In the second case, the CMTC-affected limb was 0.2 cm shorter and had circumference differences of 1.1 cm for the midthigh and 0.7 cm for the midcalf.

### Risk factors and prognosis

#### Impact of environmental and physiological factors

Eleven patients (50%) reported some response to environmental or physiological changes (Table 2). Among them, nine patients had lesions that did not resolve with warming.^11–13,15–17,25^ Cold exposure exacerbated lesions in two patients.^12^ Additionally, exacerbations were triggered by crying in one patient,^18^ fever in one patient^12^ and exercise in another.^26^ The remaining 11 patients did not report a specific response to environmental or physiological factors.

#### Fading over time

Monitoring of CMTC was documented in seven patients (32%). Three patients had lesions that remained stable over varying periods: four months,^15^ 12 months,^12^ and two years.^12^ Three patients showed spontaneous improvement, with fading or partial resolution of lesions, within three months,^17^ four months,^10^ and six months.^14^ Additionally, one patient had significant improvement of CMTC following intervention with pulsed dye laser (PDL) and intense pulsed light (IPL) over the course of 18 months^24^ (Table 2).

### Comorbidities

Half of the patients had a comorbid disorder. Of these 11 patients, six (55%) had ophthalmologic involvement, two (18%) had cutaneous involvement, and three (27%) had systemic involvement.

#### Ophthalmologic

Six cases (27%) reported ophthalmologic defects (Table 2). Half of these patients had facial involvement of CMTC. Bilateral neovascularization and peripheral retinal nonperfusion were observed in two patients.^19,28^ Bilateral peripheral nonperfusion and enlargement of the foveal avascular zone were noted in two patients, one of whom also had bilateral incomplete peripheral vascularization.^20^ Avascularity, neovascularization, venous dilations, and shunts in the temporal and superior retina were observed in one patient.^21^ Bilateral diffuse ocular melanocytosis with unilateral peripheral retinal avascularity and ischemic retinopathy was reported in one patient.^22^

#### Cutaneous

Two patients had associated cutaneous anomalies (Table 2). One patient had a solitary cutaneous mastocytoma, and another had two infantile hemangiomas.^12^

#### Systemic

Systemic involvement was observed in three patients (Table 2). One patient presented with slight elevations in aspartate aminotransferase and lactate dehydrogenase following a liver function test, indicating liver function abnormalities and potential liver damage.^14^ One patient had Rubinstein-Taybi syndrome (RSTS), presenting with microcephaly, arched eyebrows, down-slanting palpebral fissures, a broad nasal bridge, broad big toes, polydactyly, and a triangular mouth, as well as cognitive and developmental delays.^15^ One patient experienced exercise-induced transient edema, a localized systemic adverse effect, in the right lower extremity.^26^

## Discussion

This review analyzed recent cases of CMTC, highlighting its variable presentation, comorbidities, and management challenges. The findings underscore the importance of early recognition and tailored management strategies for this rare condition.

Patients ranged from neonates to adolescents, with a slight predominance of females. The majority (55%) of lesions were localized, most commonly to the lower extremities; 41% had a generalized distribution. Atrophy, limb asymmetry, and ulceration were observed in 41%, 32%, and 18% of cases, respectively, with circumference discrepancies due to hypotrophy being the most common type of asymmetry. CMTC lesions remained stable or spontaneously resolved in most cases. Half of the patients had associated comorbidities. Although vascular anomalies and neurologic defects (e.g., neurodevelopmental delays) have been described in the literature,^1,29^ none of the 22 cases presented with any. Most of the comorbidities identified in this review were ophthalmologic (e.g., neovascularization and retinal nonperfusion), with the remaining being cutaneous (e.g., infantile hemangioma) or systemic (e.g., liver function abnormalities).

An assessment of the quality of studies summarized in this review was conducted using the JBI Critical Appraisal Checklists for Case Reports and Case Series. These checklists evaluated different factors, with the case series checklist^8^ (Supplementary Table S1) considering inclusion criteria, participant and clinic demographics, and follow-up visits, while the case report checklist^9^ (Supplementary Table S2) focused on patient history, diagnostic tests, and takeaway lessons. Most studies were scored above the median, indicating moderate-to-high quality and a low risk of bias (Table 1). However, one study received a lower quality rating, corresponding to a moderate risk of bias. Items related to interventions or treatment protocols in the observational studies were marked as “not applicable” and scored as “no” which contributed to a lower overall rating of the studies.

### Clinical implications

The identification of comorbidities in 50% of cases emphasizes the importance of a multidisciplinary approach to care. Ophthalmologic complications, such as retinal nonperfusion and neovascularization, were the most frequent, occurring in 27% of cases. These findings suggest that regular ophthalmologic evaluations are critical, even for patients with no apparent ocular symptoms at the time of diagnosis. Annual follow-ups are recommended to monitor intraocular pressure and assess the progression of any complications.^2^ Additionally, the presence of systemic anomalies, such as RSTS and liver dysfunction, highlights the need for broader diagnostic evaluations in some cases.

CMTC has both psychological and physical ramifications that can significantly affect patients’ well-being. The presence of visible skin lesions, particularly in generalized cases, can lead to distress, self-consciousness, and social stigma, impacting emotional development and quality of life. Additionally, physical manifestations such as limb asymmetry, ulceration, and atrophy may not only cause discomfort and mobility challenges but also contribute to long-term functional impairments if left unmanaged. Recognizing these dual aspects of CMTC underscores the need for a comprehensive approach that includes early diagnosis to mitigate physical complications, alongside psychological support and counseling to help patients and families cope with the emotional burden of the condition.

### Treatment strategies

Management of CMTC should be guided by the severity of symptoms and associated complications, ensuring an individualized treatment plan for each patient (Table 3).

**Table 3:** Management Strategies for CMTC.

Management approach	Indication	Interventions
Conservative monitoring	Mild cases, stable lesions	Regular follow-ups
Wound care	Ulcerations	Dressings, infection prevention
Laser therapy	Significant lesions of cosmetic concerns	PDL, IPL
Limb asymmetry management	Discrepancies affecting mobility	Shoe lifts, epiphysiodesis
Ophthalmologic monitoring	Retinal or ocular involvement	Yearly ophthalmologic exams

While conservative approaches are sufficient for stable or mild cases; interventions such as laser therapy have demonstrated efficacy in improving lesion appearance and potentially preventing ulceration.^24^ For more severe cases, such as skin ulcerations, wound care is essential to prevent infection and promote healing. In cases where patients have significant limb asymmetry, early physical therapy and, in severe cases, surgical correction can optimize functional outcomes. For optimal care, a multidisciplinary approach involving dermatologists, orthopedists, ophthalmologists, and physical therapists is recommended.

Management of limb asymmetry in CMTC depends on whether the discrepancy involves length or circumference. Mild leg length discrepancies can often be managed with nonsurgical treatments, such as shoe lifts or orthotics, to improve alignment and function.^2,30^ More severe cases may require surgical interventions, such as epiphysiodesis or limb length adjustments (either internal vs. external, shortening or lengthening). Circumference discrepancies are best managed through physical therapy to strengthen the affected limb and improve functionality. Patients should be regularly monitored for limb growth and proportionality until skeletal maturity is reached.^2,30^

### Limitations and future directions

This review is limited by its reliance on case reports and case series, which, while informative, may not capture the full spectrum of CMTC presentations and outcomes. For instance, case series from 2015 to 2018^31^ reported retinal detachment, granular pigmentary abnormalities, and glaucoma among patients with CMTC—features not observed in the cases reviewed in this manuscript. Similarly, a comprehensive review spanning 1922–2019^1^ reported that approximately 5% of patients had cardiovascular defects, highlighting the variability of CMTC presentations and the potential underrepresentation of certain comorbidities in smaller studies. Conversely, there may also be a reporting bias in the opposite direction, where cases of CMTC without comorbidities are underreported. As a result, this could lead to an overrepresentation of CMTC patients with ophthalmologic, systemic, or other comorbidities in the literature, thereby limiting the generalizability of findings from case-based reviews. Moreover, while visceral anomalies have been described in some cases, a review by *Downey* et al. concluded that they are rare and unlikely to be truly associated with CMTC^32^; additional studies are needed to clarify whether these findings are incidental.

A prior review found that 88.2% of patients with generalized CMTC met the criteria for Adams-Oliver Syndrome (AOS),^32^ an association that was not systematically examined in our case selection. This raises the possibility that AOS may be underrecognized in CMTC patients, particularly those with generalized lesions.

The lack of long-term follow-up data in most reports impedes our understanding of the natural progression of CMTC and its potential late complications. Additionally, the absence of controlled studies limits the evidence base for management strategies, underscoring the need for more robust research.

Future research should focus on multicenter cohort studies to better characterize CMTC’s epidemiology, natural history, and response to treatment. Prospective studies evaluating the long-term impact of early interventions, such as laser therapy and surgical management, would provide valuable insights into optimizing care. Moreover, investigations into the genetic and molecular basis of CMTC could elucidate its pathogenesis and inform targeted therapies.

## Conclusion

CMTC is a rare but impactful condition requiring a multidisciplinary approach to management. By addressing the physical, functional, and psychosocial aspects of care, clinicians can improve outcomes for affected individuals. Increased awareness and robust clinical studies are needed to advance our understanding and management of this complex disorder.

## Supplementary information


Supplementary TableS1
Supplementary TableS2

