# Supplementary material for: Clinical presentation, risk factors, and comorbidities of cutis marmorata telangiectatica congenita: a systematic review
Source: Pediatr Res. 2025 Dec 9;100(1):120–5. doi: 10.1038/s41390-025-04588-8 (PMC13524818; doi:10.1038/s41390-025-04588-8)
Supplement: Supplementary file 1 [file 41390_2025_4588_MOESM1_ESM.pdf]

| | Leung et al., 2020 | Moreno-Alfonso et al., 2024 | Shah et al., 2024 |
|---|---|---|---|
| Sample size | 2 | 2 | 2 |
| Were there clear criteria for inclusion? | Yes | Yes | Yes |
| Was the condition measured in a standard, reliable way? | Yes | Yes | Yes |
| Were valid methods used for identification of the condition? | Yes | Yes | Yes |
| Did the case series have consecutive inclusion of participants? | N/A | N/A | N/A |
| Did the case series have complete inclusion of participants? | N/A | N/A | N/A |
| Was there clear reporting of the demographics of the participants? | Yes | Yes | Yes |
| Was there clear reporting of clinical information of the participants? | Yes | Yes | Yes |
| Were the outcomes or follow up results clearly reported? | No | Yes | No |
| Was there clear reporting of the presenting site(s)/clinic(s) demographic information? | No | Yes | Yes |
| Was statistical analysis appropriate? | No | No | No |
| Overall Score | 5/10 | 7/10 | 6/10 |

**Supplemental table S1.** JBI Checklist for case series.
