# Supplementary material for: Clinical presentation, risk factors, and comorbidities of cutis marmorata telangiectatica congenita: a systematic review
Source: Pediatr Res. 2025 Dec 9;100(1):120–5. doi: 10.1038/s41390-025-04588-8 (PMC13524818; doi:10.1038/s41390-025-04588-8)
Supplement: Supplementary file 2 [file 41390_2025_4588_MOESM2_ESM.pdf]

Study	Were the patient's demographic characteristics clearly described?	Were the patient's history clearly described and presented as a timeline?	Was the current clinical condition of the patient on presentation clearly described?	Were diagnostic tests or methods clearly described?	Was the intervention(s) or treatment procedure(s) clearly described?	Was the post-intervention clinical condition clearly described?	Were adverse events (harms) or unanticipated events identified and described?	Does the case report provide takeaway lessons?	Overall Score
Portal Buenaga et al., 2024	Yes	No	Yes	Yes	N/A	N/A	N/A	No	3/8
Kyriakou et al., 2020	Yes	Yes	Yes	Yes	N/A	N/A	N/A	Yes	5/8
Manna, 2024	Yes	Yes	Yes	Yes	N/A	N/A	N/A	Yes	5/8
Yang et al., 2023	Yes	Yes	Yes	Yes	N/A	N/A	N/A	Yes	5/8
Cardis et al., 2020	Yes	No	Yes	Yes	N/A	N/A	N/A	Yes	4/8
MacGibeny et al., 2020	Yes	Yes	Yes	Yes	N/A	N/A	N/A	Yes	5/8
Ghotbabadi et al., 2024	Yes	Yes	Yes	Yes	N/A	N/A	N/A	Yes	5/8
Kim and Kim, 2022	Yes	Yes	Yes	Yes	N/A	N/A	N/A	Yes	5/8
Hokazono et al., 2024	Yes	Yes	Yes	Yes	N/A	N/A	N/A	Yes	5/8
Rosignoli et al., 2022	Yes	Yes	Yes	No	N/A	N/A	N/A	Yes	4/8
Khambati et al., 2021	Yes	Yes	Yes	Yes	N/A	N/A	N/A	No	4/8
Tracey et al., 2020	Yes	Yes	Yes	No	N/A	N/A	N/A	Yes	4/8
Friedman et al., 2023	Yes	Yes	Yes	Yes	N/A	N/A	N/A	Yes	5/8
Belgemen-Ozer, 2024	Yes	Yes	Yes	Yes	N/A	N/A	N/A	Yes	5/8
Haidari et al., 2020	Yes	Yes	Yes	Yes	N/A	N/A	N/A	Yes	5/8
Sassalos et al., 2021	Yes	Yes	Yes	Yes	N/A	N/A	N/A	Yes	5/8

Supplemental table S2. JBI Checklist for case reports.
